# Beyond floral initiation: the role of flower bud dormancy in flowering time control of annual plants

**DOI:** 10.1093/jxb/erae223

**Published:** 2024-05-25

**Authors:** Steven Penfield

**Affiliations:** Department of Crop Genetics, John Innes Centre, Norwich Research Park, Norwich NR4 7UH, UK; University of Western Australia, Australia

**Keywords:** *Brassica*, bud dormancy, chilling, flowering, FLOWERING LOCUS C, flowering time, life history, phenology, temperature, vernalization, winter annual

## Abstract

The phenology of temperate perennials, including the timing of vegetative growth and flowering, is well known to be controlled by seasonal dormancy cycles. Dormant structures are known as buds and have specialized covering structures, symplastic isolation from the plant, and often autonomous stores of carbon and nitrogen reserves. In contrast, in annual plants, our current understanding of the control of the timing of flowering focuses on the mechanisms affecting floral initiation, the transition from a vegetative apical meristem to a inflorescence meristem producing flower primordia in place of leaves. Recently we revealed that annual crops in *Brassicaceae* exhibit chilling-responsive growth control in a manner closely resembling bud dormancy breakage in perennial species. Here I discuss evidence that vernalization in autumn is widespread and further discuss its role in inducing flower bud set prior to winter. I also review evidence that flower bud dormancy has a more widespread role in annual plant flowering time control than previously appreciated.

## Teaser

Here I introduce the importance of flower bud dormancy in the control of flowering time in annual plants, and the implications for understanding the effects of changing climates on crop production.

## Introduction

Plants adapt to seasonal environments through the evolution of timing mechanisms that optimize growth and development to occur at specific times of year, known collectively as plant phenology. In annual plants these processes include seasonal loss of seed dormancy and seed germination, the transition through juvenility to adulthood, and various stages of flowering and seed set. The timing of flowering relative to the growing season is crucial because later steps in plant reproductive development, especially male meiosis and seed development, can have specific temperature optima for optimal plant performance (Springthorpe and Penfield, 2015; Draeger and Moore, 2017; Nasahzadeh and Ellis, 2017; Lloyd *et al.*, 2018). Perennial plants, on the other hand, are able to maintain vegetative and reproductive structures simultaneously and use seasonal cycles of bud set, dormancy, and bud break to align development with the annual cycle (Yamane *et al.*, 2023). Understanding these processes is important for predicting the effects of change climate on agriculture and natural ecosystems.

For many perennial species, floral initiation occurs in spring or summer, with a dormant flower bud produced above- or below-ground that will flower in the following spring. For instance, below-ground apical meristems including bulbs such as *Tulipa gesneriana* will set flower buds in late spring or summer in response to increasing warmth (Leegangers *et al*., 2017). These basic aspects of flowering time control are broadly conserved across the *Liliaceae*, which then require winter chilling to promote bolting and flowering. The same is true of woody perennials such as sweet cherry which undergo floral initiation in mid-summer and enter dormancy before the beginning of autumn (Vimont *et al.*, 2019). The herbaceous perennial strawberry (*Fragaria* sp.) sets buds in autumn (Samad *et al.*, 2017). These limited examples show that regardless of habit, bud set prior to winter in response to changes in either temperature, photoperiod, or light quality is a common strategy in perennial angiosperms, and the underlying molecular mechanisms are broadly conserved in gymnosperms, suggesting an origin >300 million years ago (Heide and Prestrud, 2005; Lagercrantz, 2009; Cooke *et al*., 2012).

Dormant buds respond to winter chilling, which renders them competent to begin growth upon receipt of warm temperature and longer day conditions in spring, known as the transition from endodormancy to ecodormancy. Bud set is promoted by the activity of MADS box transcription factors closely related to *SHORT VEGETATIVE PHASE* (*SVP*), including *DORMANCY ASSOCIATED MADS* (*DAM*) genes and *FLOWERING LOCUS C* (*FLC*) orthologues, depending on the species (Bielenberg *et al*., 2008). These genes are down-regulated during winter, and this down-regulation probably plays a role in the release of endodormancy because ectopic expression can delay bud break (Wu *et al.*, 2017). A largely overlapping gene set governs the promotion of flowering by vernalization in annuals. However, studies of flowering time in annuals generally assume that by understanding the control of floral initiation one can learn about the control of flowering time itself, an approach that would be flawed in perennials in which dormant flower buds can be set many months before flowering itself. A key question therefore is whether bolting and flowering in annuals can be separated in time and, if so, whether the time between floral initiation and flowering contains additional growth checkpoints responsive to environmental signals that could be considered synonymous with bud break. If so, then in annual plants too understanding the control of floral initiation will only be part of the story of the control of flowering itself.

## Winter annual life history and the timing of floral initiation

In the field, winter annual *Arabidopsis thaliana* accessions have generally completed vernalization by the end of autumn (Duncan *et al.*, 2015), and at this time the vegetative apex begins floral initiation (Lu *et al.*, 2022). The temperature during autumn is ideal for vernalization, given that laboratory studies of the temperature range that permits vernalization show an upper limit in *A. thaliana* of ~14–16 °C (Wollenburg and Amasino, 2012; Hepworth *et al.*, 2018). This timing of vernalization in autumn is likely to be common place in angiosperms because research in cereals, *Brassica*, and other species consistently shows that the upper temperature limit for vernalization is ~16 °C (Tommey and Evans, 1991; Brooking, 1996; Wollenburg and Amasino, 2012).

Interestingly, although well known as a rapid cycling *A. thaliana*, the preferred laboratory accession Columbia-0 will grow with a winter annual life history if germinated in Europe in autumn (Wilczek *et al.*, 2009). Furthermore, secondary dormant Columbia seeds exhumed from the soil seed bank have both spring and autumn germination flushes, showing that Columbia is adapted to both a winter annual and summer annual life history in the field, even though it lacks a strong *FRIGIDA* (*FRI*) allele (Springthorpe and Penfield, 2015). This may be because in rapid cycling Arabidopsis accessions, lower ambient temperatures increase *FLC* expression, an increase that can be subsequently reduced again by vernalization (Chen and Penfield, 2018).

**Fig. 1.**
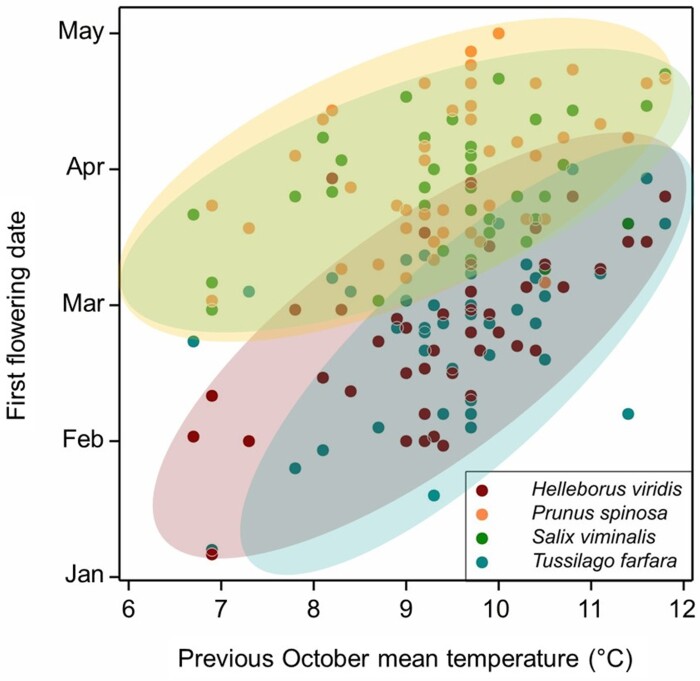
Autumn vernalization found in winter annual Brassicas is shared with perennial species native to the UK. Data show the relationship between autumn warming and spring flowering delay in two herbaceous perennials (*Helleborus* and *Tussilago*) and two woody perennials (*Salix* and *Prunus*). Data re-analysed from Fitter *et al.* (1995).

In *Brassica napus*, *FLC* genes are present in up to nine copies (Schiessl *et al*., 2015; Calderwood *et al*., 2021), each with distinct vernalization characteristics that vary by variety. In winter oilseed rape, up to five of these are reduced in expression by vernalization in autumn in the UK, prior to floral initiation during November (late autumn; O’Neill *et al.*, 2019). Thus the Arabidopsis and *Brassica* genera have in common the presence of *FLC* genes with autumn vernalizing characteristics. We could confirm using field experiments that warming winter oilseed rape in autumn delays flowering the following spring, consistent with interruption of chilling. Interestingly, in a dataset containing flowering dates of 243 angiosperm species over 36 years in central England, a large number were retarded in spring flowering by warmer temperatures in the preceding autumn (Fitter *et al.*, 1995). For many of these lines, flowering time the following spring correlates positively with the temperature during the previous October (Fig. 1), including both herbaceous and woody perennials. In this study, a generalized difference in the effect of autumn warming was observed with spring flowering lines enriched for those in which warming delays flowering, and later summer flowering lines enriched for those in which autumn warming advances flowering (Fitter *et al.*, 1995). The warming-delayed set included annuals, and herbaceous and woody perennials; therefore, these characteristics of plant phenology are largely conserved across species with contrasting life histories. Autumn warming probably also delays vegetative bud set in woody perennials and may buffer forest phenology against the effects of climate change by delaying spring bud burst during warmer growing seasons (Heide, 2003).

Short-lived perennials in the *Brassica* family also probaby display a similar phenology of autumn vernalization followed by bud set in late autumn/early winter. In *Arabidopsis halleri* growing in Japan, *FLC* transcripts have been substantially reduced by vernalization before the beginning of winter, and expression of the inflorescence meristem marker gene *SUPPRESSOR OF CONSTANS 1* (*SOC1*) can be detected at the beginning of winter (Aikawa *et al.*, 2010). This indicates that *A. halleri* plants have also undergone the floral transition in late autumn. A similar phenology can probably be found in *Arabis alpina* because, like *B. napus*, flower bud set can be observed during vernalization treatments, rather than after subsequent transfer to warm long days (Wang *et al.*, 2009). Taken together, these findings clearly show that the autumn vernalization and floral initiation at cold temperatures are observed in many species of the *Brassica* family, regardless of whether they show a winter annual or perennial habit.

## Dormant flower bud set in winter *B. napus* requires floral initiation in short days

These observations contrast with classical models of the control of flowering time in which vernalization in winter enables the longer warmer days in spring to promote floral initiation in spring. After vernalization, floral initiation can in principle be promoted by two distinct processes. Firstly, vernalization in leaves leads to the loss of repression of *FLOWERING LOCUS T* (*FT*) and translocation of the FT protein to the apical meristem (Corbesier *et al*., 2007). At the apex itself, *SOC1* is a direct target of FLC and is activated by loss of FLC repression to promote floral initiation. However, *FT* expression in both *B. napus* and *A. halleri* is not observed until much later in winter, closer to the onset of bolting than floral initiation (Aikawa *et al*., 2010; O’Neill *et al.*, 2019). Thus, given that FT is also expressed during bolting, bolting and bud break are likely to be analogous processes. Floral initiation in late autumn necessarily means that it takes place in short days in the absence of high levels of FT activity. Viewed in this way, a requirement for autumn vernalization acts mainly to delay floral initiation until the day length is too short and the ambient temperature too cool to promote bolting. In arable winter annual *Brassica* crops, the timing of floral initiation relative to the winter equinox may be important for yield potential because weak alleles of *FT* are associated with higher plant yields in the species (Raman *et al.*, 2019).

In Arabidopsis, flowering in short days has an absolute requirement for gibberellic acid (GA) to promote both *LEAFY* and *SOC1* expression at the shoot apex (Wilson *et al.*, 1992; Eriksson *et al.*, 2006). GA_4_ content at the apex increases prior to flowering in short days, but it remains unclear how this increase is achieved. The basic helix–loop–helix (bHLH) transcription factor gene *NO FLOWERING IN SHORT DAYS* (*NFL*) is likely to be important for GA-induced flowering because mutants have a non-flowering phenotype only when grown in short days, and this is rescued by exogenous GA application (Sharma *et al.*, 2016). The precise timing of floral initiation in short days in Arabidopsis is also affected by the expression level of *TERMINAL FLOWER 1* (*TFL1*) which delays flowering and is regulated by CONSTANS in the shoot apex in a manner paralleling the regulation of *FT* in phloem (Koskela *et al*., 2012; Luccioni *et al.*, 2019).

## Evidence for flower bud set in annual plants

Buds are characterized by the presence of partly differentiated vegetative or reproductive structures either at the main apex or on axils of leaves. Most commonly they are enclosed by specific structures which exhibit protective characteristics such as waxy or suberized cuticles. However, a key feature is the potential for growth arrest and quiescence requiring inhibition or slowing of cell division and growth, coupled with the requirement for either endogenous or environmental signals for further development (Considine and Considine, 2016; Velappan *et al.*, 2017; Fadón *et al*., 2019). Buds may be isolated from the vascular system of the plant and simplastically by callose formation in plasmodesmata (Rinne *et al.*, 2011). In *A. thaliana* lateral buds, FT and BRANCHED1 (BRC1) act to coordinate bud growth, with BRC1 antagonizing the growth-promoting activity of FT (Niwa *et al.*, 2013). This process is substantially conserved during seasonal growth cessation in poplar, with short photoperiods activating *BRC1* expression which acts to suppress growth, in part by antagonizing residual FT protein function (Maurya *et al.*, 2020). In some species, TFL1 also plays a clear role in opposing FT activity: the *Rosaceae* are a good example of this where loss of TFL1 prevents flower bud dormancy and results in a perpetual flowering phenotype (Iwata *et al.*, 2012).

Although an overlapping gene set controls bud outgrowth and flower initiation in plants, a hallmark of perennial behaviour is the temporal uncoupling of flower bud set from bolting or bud break and flowering itself. Furthermore, perception of one or more environmental signals is required to pass through at least one stage in flower development, with that stage being variable between species, but perhaps not between cultivars of the same species (Fadón *et al*., 2019).

**Fig. 2.**
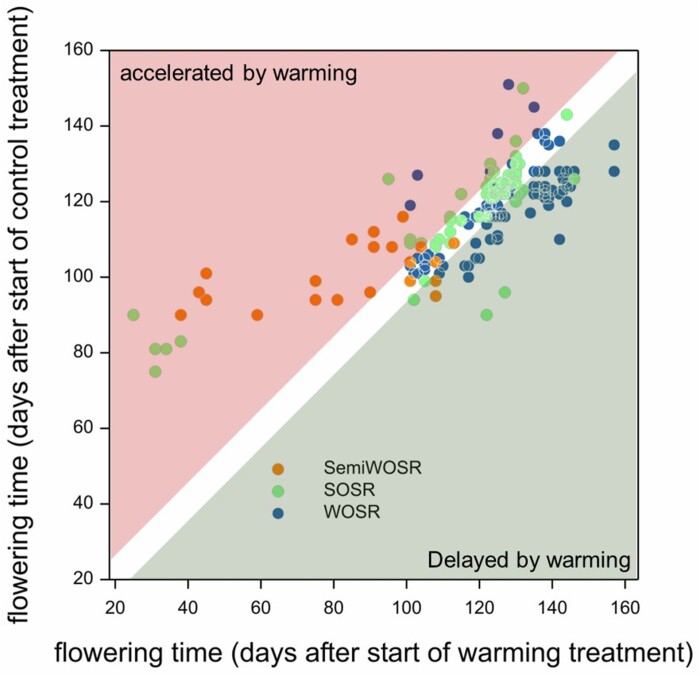
*Brassica napus* flower bud responses to winter temperature variation in short days depend on crop type. Spring oilseed rape (SOSR), winter OSR (WOSR), and Chinese semi-winter OSR have distinct flowering time responses to warming in short days, reflecting adaptation by breeding. Data extracted from Lu *et al.* (2022).

In our work to uncover sources of yield variation in winter oilseed rape, we found that expression of at least two of the nine *FLC* genes was not responsive to vernalization that occurs prior to floral initiation. This led us to hypothesize that these genes had an additional role in developing reproductive tissues. In *B. napus*, well-vernalized winter annual varieties will flower swiftly after transfer to warm long days (Schiessl *et al.*, 2015). However, we found that if vernalized plants that have undergone floral initiation are placed in warm short days, bolting and flowering are instead delayed relative to chilling treatments (Lu *et al.*, 2022). Thus, in short days, the normal relationship between increasing temperature and reproductive development can be reversed. Analysis of the effects of temperature showed that warming retarded flower bud growth and strongly inhibited expression of cell cycle-related genes. Furthermore, warming caused elevated abscisic acid (ABA) levels in flower buds and increased expression of genes known to be involved in the ABA repression of axillary bud outgrowth in Arabidopsis when their outgrowth is inhibited by far-red light (González-Grandío *et al.*, 2017). Together, these observations strongly suggest that the combination of short days with lack of winter chill induces flower bud dormancy in *B. napus* (Lu *et al*., 2022). This combined effect of short days and warmth was only observed in winter crop types and was affected by variation in specific *FLC* genes that are silenced by winter chilling of flower buds. Interestingly, Chinese semi-winter varieties adapted to the short winters of the Yangtze River Basin could proceed to flower in warm short days, whereas flower buds of European and North American spring varieties required longer days to promote bolting and flowering but were on average unaffected by temperature variation (Fig. 2). Taken together, this shows that in the annual plant *B. napus*, temperature and day length signals interact to promote growth cessation and the timing of bolting by acting after floral initiation on developing flower buds.

In winter wheat, flower bud set is commonly known as spike development, and occurs in day-neutral conditions around the spring equinox. For winter wheat growing in the UK, different flower developmental stages appear to have differing critical photoperiods. For instance, progression to early inflorescence development occurs in day lengths exceeding 11.5 h which promote a small rise in *FT1* expression. However, if plants remain at this photoperiod, further floral development stalls (Gaulay and Boden, 2021). Longer day lengths promote later stages of development by promoting higher *FT1* transcript levels, and also via activation of a second wheat *FT* orthologue, *FT2*. These observations demonstrate the existence of a photoperiod-responsive developmental checkpoint during floral development in cereals that resembles the manner in which long days overcome ecodormancy during the initiation of perennial bud burst. They also show that functional diversification of the *FT* gene family can be co-opted by evolution to generate a number of critical photoperiods required for progression beyond sequential developmental stages.

## Understanding flower developmental stages that permit flower bud dormancy in the *Brassicaceae*

At the molecular level, the *Brassica* family remains the best understood in terms of the mechanism for the control of reproductive development. During the early stages of flower development, undifferentiated flower primordia are initiated from the inflorescence meristem (Stages 1 and 2). In *A. thaliana* at this stage, *SVP*, *AGAMOUS-LIKE 24* (*AGL24*), and *SOC1* are expressed in flower primordia, and alongside APETALA1 (AP1), act to repress floral homeotic gene expression necessary for floral organ differentiation (Gregis *et al*., 2008, 2009). Given that transcription factors closely related to SVP act to promote bud dormancy in perennials, this stage is an obvious candidate for the manifestation of quiescence in annual plants. In *B. napus* during the time at which we observe flower bud dormancy, levels of *TFL1*, *SVP*, *SOC1*, and *AGL24* transcripts remain high and are only replaced by *FT*, *AP1*, and other floral homeotic genes as the plants begin to bolt in late winter (O’Neill *et al.*, 2019). Insufficient chilling also permits *FLC* expression necessary for the manifestation of bud dormancy at the same developmental stage (Lu *et al.*, 2022). In *A. thaliana* there is so far little evidence for a developmental checkpoint post-floral initiation in wild-type plants. However, it may be noteworthy that flower buds arrested at this early stage can be found in double mutants lacking *AP1* and *CAULIFLOWER* (*CAL*; Bowman *et al*., 1993), or *AP1*, *AGL24*, and *SVP* (Gregis *et al.*, 2008). These plants bolt but produce inflorescences with extra branched inflorescence meristems and in which a considerable delay occurs before floral development resumes.

However, there appears to be no generalizable rule to the stage of floral development at which bud dormancy can occur. In *Brassica oleracea*, flower bud arrest can take place at different developmental stages, leading to the production of cauliflowers or different forms of calabrese or broccoli if some floral organ differentiation has taken place. Although cauliflower curds arrest at an apparently similar developmental stage to *ap1 cal* buds, the mechanism of arrest appears distinct because most cauliflower varieties contain functional *CAL*, *AP1*, *SOC1*, *SVP*, and *AGL24* genes. Instead, genomic analysis suggests that multiple alterations to the MONOPTEROUS/auxin-regulated floral initiation process are found specifically in cauliflower varieties rather than in other *B. oleracea* crop types (Guo *et al.*, 2021). Once floral initiation has begun, cauliflower curd development remains temperature sensitive: temperatures colder than optimum promote the phenomenon of riciness, associated with premature initiation of floral organ primordia (Fujime and Okuda, 1996). This appears to show that chilling developing curds can accelerate floral development, suggesting that chilling-responsive growth inhibitors act to maintain curd identity in cauliflowers.

This shows that arrest itself can occur at different stages of flower development. A similar conclusion is supported by studies in cherry which showed that flower bud dormancy occurred when floral organs were fully differentiated and male and female reproductive organs had completed full organogenesis, but before meiosis was complete (Fadón *et al.*, 2019). So it remains likely that expression patterns of chilling-responsive transcription factors differ between and within species, resulting in observable dormancy in different developing flower structures. In *B. napus*, we can even detect differential chilling responses between different floral organs in the same flower, with warming having a greater effect on petal and anther development than on carpels or sepals (Lu *et al.*, 2022).

## Conclusions

**Fig. 3.**
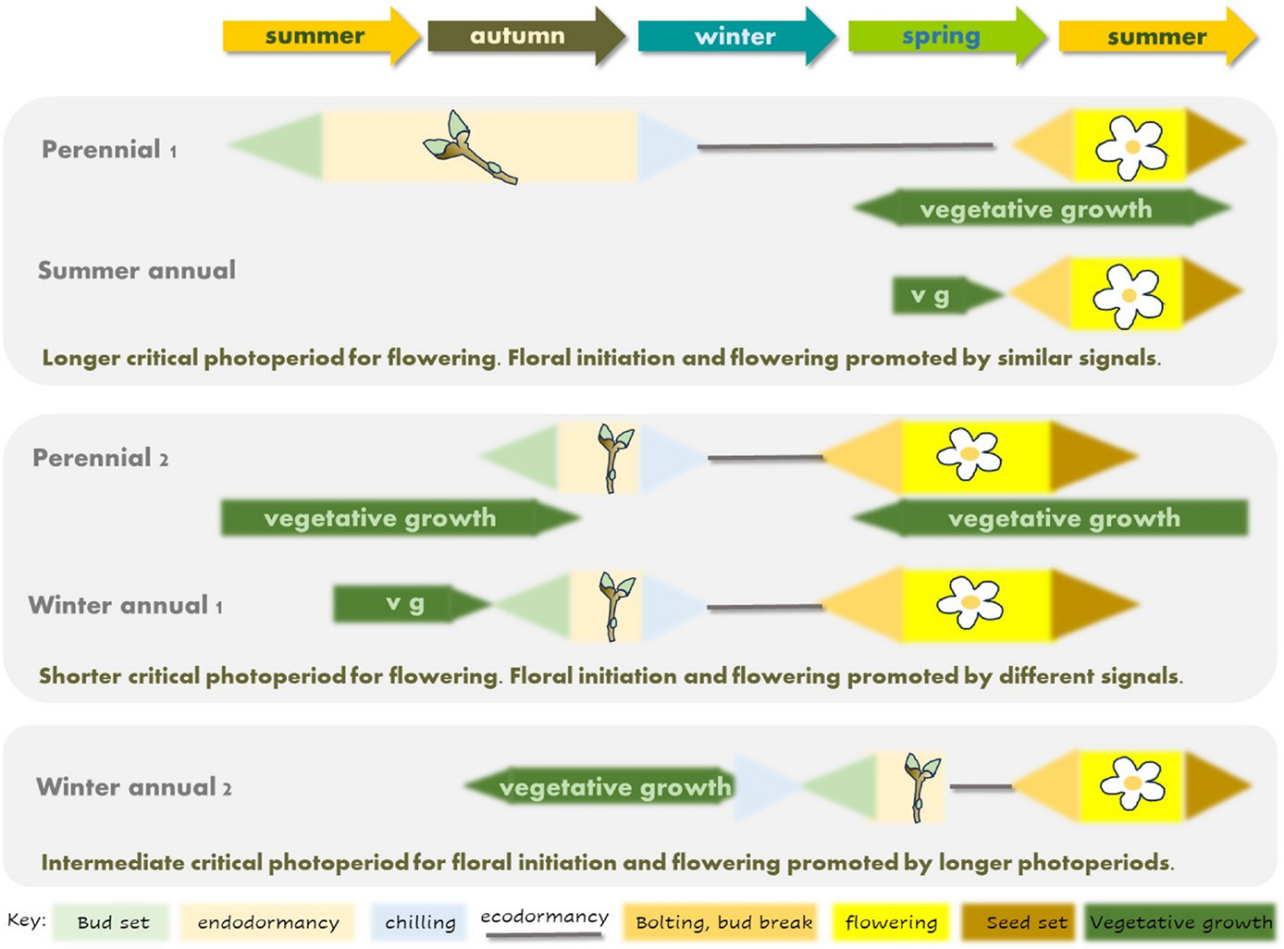
Similarities between the role of bud dormancy in perennial phenology and annual plant phenology. Summer annuals and some perennials share the fact that floral initiation and flowering occur at similar times of year, although in perennials the two processes occur in different calendar years. Winter annuals can share phenological features with perennials that set flower buds in autumn, probably those whose flowering is delayed by autumn warming (Fitter *et al.*, 1995). Winter annuals such as wheat experience chilling during the vegetative phase and thus do not exhibit a flower bud endodormancy-like syndrome, but nevertheless there is evidence of reproductive checkpoints which parallel ecodormancy in perennials such as photoperiod responsiveness. Vg, vegetative growth.

The widespread link between autumn warming and a delay to spring flowering (Fitter *et al.*, 1995) suggests that responses to chilling in autumn are widespread in flowering plants, regardless of life history. This behaviour contrasts with a second class of summer flowering species whose flowering is on average advanced by warming. One possibility is that annual and perennial species share aspects of phenology control, dividing broadly into two classes (Fig. 3). The first, comprising summer flowering perennials and summer annuals, shares the timing and environmental signals that promote both floral initiation and flowering, but perennials in this class differ from summer annuals in that they have a long bud dormancy phase such that floral initiation and flowering occur in different growing seasons. Summer annuals may nevertheless exhibit a type of ecodormancy which, as in the case of spring oilseed rape, requires a photoperiod signal to initiate bud burst, bolting, and flowering (Lu *et al.*, 2022). This is not usually observed in experiments because laboratory processes which promote the transition to flowering also break the ecodormancy-like requirement for an environmental signal to initiate bolting. A second class comprises winter annuals and spring-flowering perennials which initiate flower buds in response to autumn vernalization. Finally, there are examples of winter annuals that set buds in spring but nevertheless require a distinct environmental signal to initiate bolting, such as winter wheat.

## Data Availability

The data underlying Fig. 1 can be found in the Supplementary files associated with Fitter *et al.* (1995). Data for Fig. 2 are reproduced from Lu *et al.* (2022).
